# Inverse-designed taper configuration for the enhancement of integrated 1 × 4 silicon photonic power splitters

**DOI:** 10.1515/nanoph-2024-0295

**Published:** 2024-09-09

**Authors:** Seokjin Hong, Jinhyeong Yoon, Junhyeong Kim, Berkay Neseli, Jae-Yong Kim, Hyo-Hoon Park, Hamza Kurt

**Affiliations:** The School of Electrical Engineering, 34968 Korea Advanced Institute of Science and Technology (KAIST), Daejeon, Republic of Korea

**Keywords:** silicon photonics, photonic power splitter, inverse design, MMI, particle swarm optimization

## Abstract

Once light is coupled to a photonic chip, its efficient distribution in terms of power splitting throughout silicon photonic circuits is very crucial. We present two types of 1 × 4 power splitters with different splitting ratios of 1:1:1:1 and 2:1:1:2. Various taper configurations were compared and analyzed to find the suitable configuration for the power splitter, and among them, parabolic tapers were chosen. The design parameters of the power splitter were determined by means of solving inverse design problems via incorporating particle swarm optimization that allows for overcoming the limitation of the intuition-based brute-force approach. The front and rear portions of the power splitters were optimized sequentially to alleviate local minima issues. The proposed power splitters have a compact footprint of 12.32 × 5 μm^2^ and can be fabricated through a CMOS-compatible fabrication process. Two-stage power splitter trees were measured to enhance reliability in an experiment. As a result, the power splitter with a splitting ratio of 1:1:1:1 exhibited an experimentally measured insertion loss below 0.61 dB and an imbalance below 1.01 dB within the bandwidth of 1,518–1,565 nm. Also, the power splitter with a splitting ratio of 2:1:1:2 showed an insertion loss below 0.52 dB and a targeted imbalance below 1.15 dB within the bandwidth of 1,526–1,570 nm. Such inverse-designed power splitters can be an essential part of many large-scale photonic circuits including optical phased arrays, programmable photonics, and photonic computing chips.

## 1 Introduction

Since the continuous evolution of photonics integrated circuits (PICs) has brought a new era in the photonics field due to their high performance, compactness, and efficiency [1], [2], a number of recent researches have focused on on-chip photonic components [3], [4], [5], [6]. These components, such as modulators, resonators, filters, and power splitters, are effectively used in many photonic applications including optical sensing [7], [8], [9], optical communication [10], [11], [12], and photonic computing [13], [14], [15]. Among them, the photonic power splitters (PSs) have established themselves as indispensable components. Various PS structures, including multimode interference (MMI) couplers [16], [17], [18], [19], [20], Y-junction [21], [22], [23], directional couplers [6], [24], [25], and photonic crystal structures [26], [27], have been proposed. Especially, MMI couplers utilizing the self-imaging principle have gained widespread adoption due to their characteristics of low loss, compact size, high fabrication tolerance, and large operating bandwidth [28].

While MMI-based PSs have been predominantly researched for the 1 × 2 configuration, there is a growing demand for PSs with additional output ports to enhance scalability [6], [16], [17], [18], [29], [30]. Even though a series of typical 1 × 2 PS trees have been used to split light into multiple channels, it has proven to be challenging due to the large system size caused by multiple stages. Simply reducing the number of stages in the PS tree can greatly help in building more compact systems. However, conventional 1 × 4 MMI-based PSs typically occupy a large footprint to ensure high performances. Consequently, recent efforts have increasingly utilized inverse design techniques to design multi-output PSs while ensuring their performance [17], [18], [30]. Inverse design is a design methodology that relies on proven optimization algorithms such as particle swarm optimization (PSO) [16], [17], [18], topology optimization [23], [24], genetic algorithm (GA) [31], [32], and direct binary search (DBS) [33], [34], [35]. Unlike traditional design methods that rely on human intuition, the inverse design utilizes these algorithms to optimize the necessary parameters for effective design. Yao et al. in ref. [17] proposed 1 × 4 MMI-based PS using inverse design. The input and output ports were divided into several segments, and the widths of each segment were optimized through PSO. Experimental results of this PS show low insertion loss and imbalance within a wide bandwidth. In ref. [18], the authors divided the MMI region of 1 × 4 PSs into multiple segments, and the PSO was utilized to find optimal values for the dimensions of divided segments. These PSs achieved low insertion loss and imbalance with compact footprints.

In this work, we propose compact inverse-designed 1 × 4 PSs that achieve high performance within a wide bandwidth. Two types of PS were designed and experimentally validated. The proposed PS structures feature a simple MMI region with parabolic tapers applied to the input and output ports. Several taper configurations were examined to find an appropriate taper shape for use in MMI-based PSs and a parabolic port was selected. Then, the dimensions of the PSs were optimized through PSO. This resulted in the design of PS with power distributed evenly among four output ports in a 1:1:1:1 ratio and PS with a ratio of 2:1:1:2. Both PSs have been fabricated on a 220-nm SOI platform with compact footprints of 12.32 × 5 μm^2^. The PS with the splitting ratio of 1:1:1:1 exhibits insertion loss of less than 0.61 dB, and imbalance of less than 1.01 dB in a bandwidth of 1,518–1,565 nm. Also, the PS with the splitting ratio of 2:1:1:2 achieves an insertion loss of less than 0.52 dB, and a targeted imbalance of less than 1.15 dB within a bandwidth of 1,526–1,570 nm.

## 2 Design and optimization

**Figure 1:**
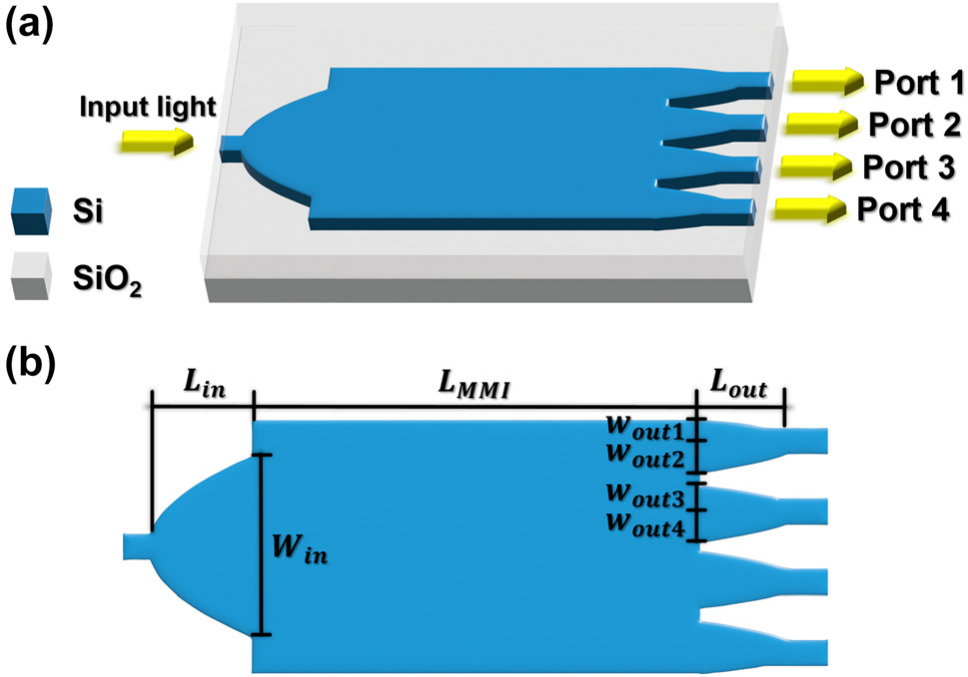
A schematic of (a) the 1 × 4 PS. (b) Design parameters utilized for optimization of both PSs with splitting ratios of 1:1:1:1 (PS-1) and 2:1:1:2 (PS-2). Two PSs with different splitting ratios can be designed by adjusting the widths of parabolic ports, *W*_in_, *W*_out,a_, *W*_out,b_, *W*_out,c_, and *W*_out,d_. Here, *W*_out,a_ and *W*_out,b_ are the half-widths of output port 1. Similarly, *W*_out,c_ and *W*_out,d_ indicate the half-widths of output port 2. The overall structure is symmetrical.

A schematic of the 1 × 4 PS proposed in this work is shown in Figure 1(a). The device features parabolic input and output ports. We aimed to design two types of PSs with different splitting ratios. The PS with a splitting ratio of 1:1:1:1 (PS-1) and the PS with a splitting ratio of 2:1:1:2 (PS-2) have the same framework as illustrated in Figure 1(a), the only differences are the widths of input and output ports. In MMI-based PS, N-folded images are generated at a specific length of MMI following the self-imaging principle [28]. Thus, the proposed 1 × 4 PSs can be designed by finding optimal parameters of MMI which form 4-folded images.

### 2.1 Selection of the taper configuration

The design of the 1 × 4 PS began with selecting a configuration of tapered ports. Conventionally, linear tapers have been widely utilized, but recent research has proposed alternative taper configurations to increase coupling efficiency and consequently reduce crosstalk [36], [37], [38]. In this section, various types of tapers were investigated to find the most effective structure for MMI-based PS. The fundamental TE mode as an input light is converted to higher-order modes within the MMI region. Then the light splitting occurs due to interference among these higher-order modes. If a short-length input taper can efficiently convert light into higher-order modes, it is possible to reduce the length of the MMI region, enabling a more compact design of the entire device.

To explore a taper configuration suitable for the transition to higher-order modes, the propagation characteristic of the fundamental TE mode was numerically calculated at a wavelength of 1,550 nm. The mode transition region is composed of a 500 nm width single-mode input waveguide and a 5 μm width multimode output waveguide. The coupling region between these two waveguides was adopted among three different configurations, parabolic, exponential, and linear tapered ports. The taper length (*L*_taper_) was set to 2 μm, and the taper output width (*W*_taper_) varied from 1 μm to 5 μm in increments of 0.5 μm.

**Figure 2:**
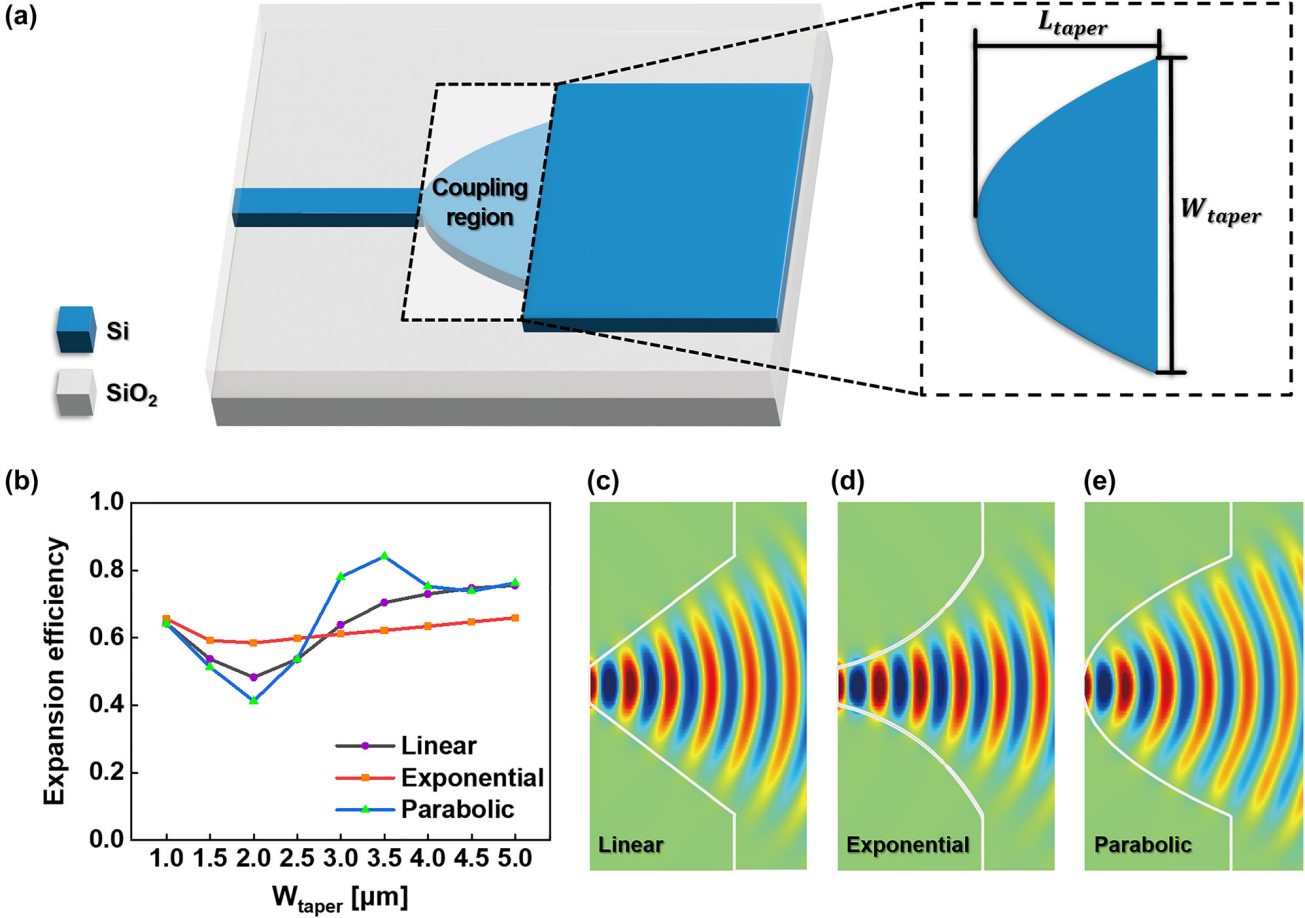
A schematic of (a) parabolic taper connecting single mode waveguide and multimode waveguide. The parabolic taper can be replaced with linear or exponential taper. (b) Expansion efficiency as a function of *W*_taper_, *E*_*y*_-field distributions of the (c) linear taper, (d) exponential taper, and (e) parabolic taper at the *W*_taper_ of 3.5 μm.

The representative example of the coupling region is illustrated in Figure 2(a). For numerical expression of the degree of conversion to higher-order modes, the modal expansion efficiency (MEE) was defined as follows:

(1) $$MEE=\frac{P_{\text{high}}}{P_{\text{tot}}},$$

where *P*_tot_ represents the total output power, and *P*_high_ denotes the output power corresponding to higher-order modes excluding the fundamental TE mode. The output power was measured in the MMI region, located 1 μm away from the final point of the input taper port. MEE becomes high when the taper converts fundamental TE mode to higher-order mode efficiently. Figure 2(b) illustrates the MEE corresponding to different types of taper. For *W*_taper_ below 2.5 μm, the MEE of the exponential taper is marginally higher compared to other tapers. From 3 μm, the *MEE* of the parabolic taper significantly increases, peaking around 3.5 μm. Figure 2(c)–(e) illustrate the propagation of the *E*_*y*_-field inside the parabolic, exponential, and linear tapers, respectively, when *W*_taper_ is set to 3.5 μm. This analysis shows that the parabolic port converts to higher-order modes efficiently, so it was selected for the design of 1 × 4 PSs.

### 2.2 Inverse design of 1 × 4 power splitters

The design parameters for optimization of the proposed 1 × 4 PSs are shown in Figure 1(b). The PSO is utilized to search the optimal lengths of MMI (*L*_MMI_), input port (*L*_in_), and output ports (*L*_out_), as well as widths of input port (*W*_in_), and output ports (*w*_out,a_, *w*_out,b_, *w*_out,c_, *w*_out,d_). PSO is an iterative optimization algorithm developed from the simulation of a simplified social model [39] and has been widely utilized in the inverse design of various photonic devices [16], [17], [18]. In PSO, particles refer to potential solutions, and the number of particles evaluated in a single iteration is equal to the population size. In the design of the 1 × 4 PSs, the sets of potential design outcomes or the set of design variables can be referred to as particles. Particles are initially randomly positioned and assigned velocities in the multidimensional space corresponding to the number of design variables. Each particle is evaluated based on a predetermined figure of merit (FOM), then the individual best position (*pb*) of each particle and the global best position (*gb*) within the entire population are recorded. Subsequently, the positions and velocities of particles are updated according to the following equations.

(2) $$v_{t+1}=\omega_{e}v_{t}+c_{1}\eta_{1}\left(pb_{t}-x_{t}\right)+c_{2}\eta_{2}\left(gb_{t}-x_{t}\right),$$

(3) $$x_{t+1}=x_{t}+v_{t+1},$$

where *x* and *v* denote the position and velocity of the particle, *ω*_*e*_, *c*_1_ and *c*_2_ represent the inertial weight, cognitive rate, and social rate, respectively. *η*_1_ and *η*_2_ are uniformly distributed random coefficients between 0 and 1. The subscript *t* represents the *t*-th iteration. This process is repeated to search for the optimal solution, and optimization is considered complete when the maximum number of iterations is reached or when a sufficient FOM is achieved.

When designing a PS-1, the width of the MMI was fixed at 5 μm, and the gap between adjacent output ports was chosen as 0.9 μm to prevent evanescent coupling. Since the power splitter is a linear device, it is possible to optimize the front and rear portions of the device sequentially. The optimization process began with searching *L*_in_, *W*_in_, and *L*_MMI_ in order to find the optimal values for dividing the input light evenly. During the optimization, *L*_in_, *W*_in_, and *L*_MMI_ varied within the ranges of [0.5 μm, 2 μm], [1 μm, 5 μm], and [5 μm, 12 μm], respectively. Afterward, *L*_out_, *w*_out,a_, *w*_out,b_, *w*_out,c_, and *w*_out,d_ were adjusted to further enhance the performance of the PS in the second optimization. *L*_out_ was allowed to move between 0.5 and 2 μm. Also, *w*_out,a_, *w*_out,b_, *w*_out,c_, and *w*_out,d_ were constrained within the interval of [0.25 μm, 0.7 μm]. Each single output port has asymmetric taper widths on its two sides, but the overall structure of the PS maintains symmetry. Organizing the optimization process into two steps reduces the number of variables used in a single optimization, thus decreasing the complexity of optimization and mitigating the problem of falling into local minima. The same FOM was minimized in two optimization processes, and the employed FOM is as follows:

(4) $$FOM=\sqrt{\overset{4}{\underset{k=1}{\sum }}\left[{\left(\frac{P_{in}}{4}-P_{k}\right)}^{2}\right]},$$

where *P*_*k*_ is the output power of the *k*-th output port, and *P*_in_ is the input power. The optimized parameters were compensated according to fabrication tolerance. As a result, the length of the MMI region was selected as 8.64 μm. In addition, the length and width of the input taper port were chosen as 2 μm and 3.54 μm, respectively. The optimal width of the input taper port closely resembles the previously mentioned *W*_taper_ value of 3.5 μm, which demonstrated the maximum MEE in Section 2.1.

The PS-2 was designed based on PS-1. Initially, the width of the parabolic taper was varied to adjust the splitting ratio. Subsequently, the widths of the output tapers were fine-tuned again. The FOM utilized in the optimization of the PS-2 is as follows:

(5) $$FOM=\sqrt{{\left(\frac{P_{in}}{3}-P_{1}\right)}^{2}+{\left(\frac{P_{in}}{6}-P_{2}\right)}^{2}+{\left(\frac{P_{in}}{6}-P_{3}\right)}^{2}+{\left(\frac{P_{in}}{3}-P_{4}\right)}^{2}}.$$

**Figure 3:**
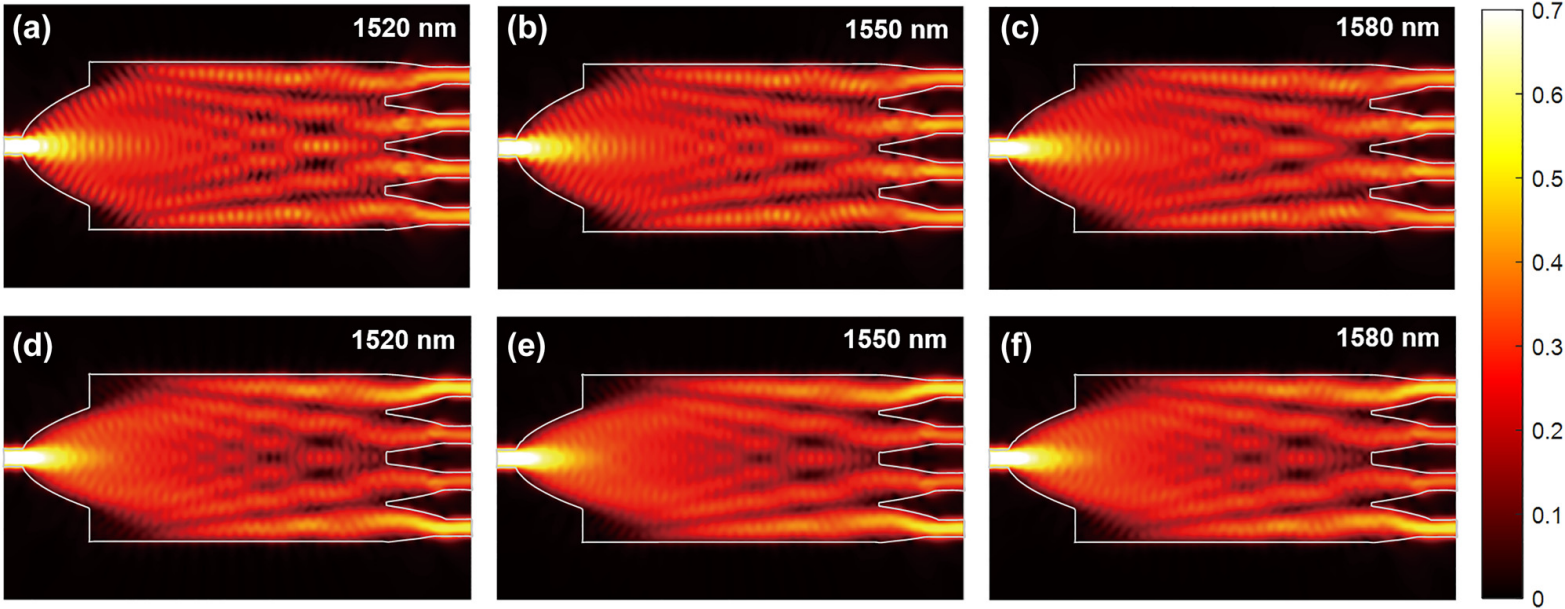
E-field distributions for the PS-1 at the wavelengths of (a) 1,520 nm, (b) 1,550 nm, (c) 1,580 nm, and for the PS-2 at the wavelengths of (d) 1,520 nm, (e) 1,550 nm, (f) 1,580 nm.

The parameters were likewise compensated after the optimization to meet fabrication tolerance. Finally, the width of the input taper was determined as 2.96 μm. The designed two types of PSs operated over a wide bandwidth covering the entire C-band and L-band, as well as a portion of the S-band. During the optimization process, 2.5D FDTD was used and subsequently optimized devices were validated with 3D FDTD. The optimization was performed over a wavelength range of 1,480 nm–1,620 nm, with the mesh size set uniformly to 20 nm in both the *x* and *y* directions. The simulation time for the optimization was approximately 12 h on a desktop computer equipped with a 2.1 GHz Core i7 CPU and 64 GB of RAM. The E-field propagations of 3D FDTD in the optimized PSs are illustrated in Figure 3. The parabolic input ports successfully convert the fundamental TE modes to multi-modes. In Figure 3(a)–(c), it can be observed that the PS-1 evenly divides the input power into the four output ports at the three different wavelength points of 1,520 nm, 1,550 nm, and 1,580 nm. Similarly, Figure 3(d)–(f) show that the PS-2 forms four images with the power ratio of 2:1:1:2 at the output ports. Both PSs achieved the desired splitting ratio within the large bandwidth. Additionally, replacing the ports of PS-1 with linear tapers while maintaining the same design parameters resulted in decreased performance in terms of insertion loss and imbalance. Furthermore, it was not possible to adjust the splitting ratio by varying the width of the input port in the linear taper configuration.

## 3 Fabrication and experimental demonstration

### 3.1 Fabrication of 1 × 4 power splitters

**Figure 4:**
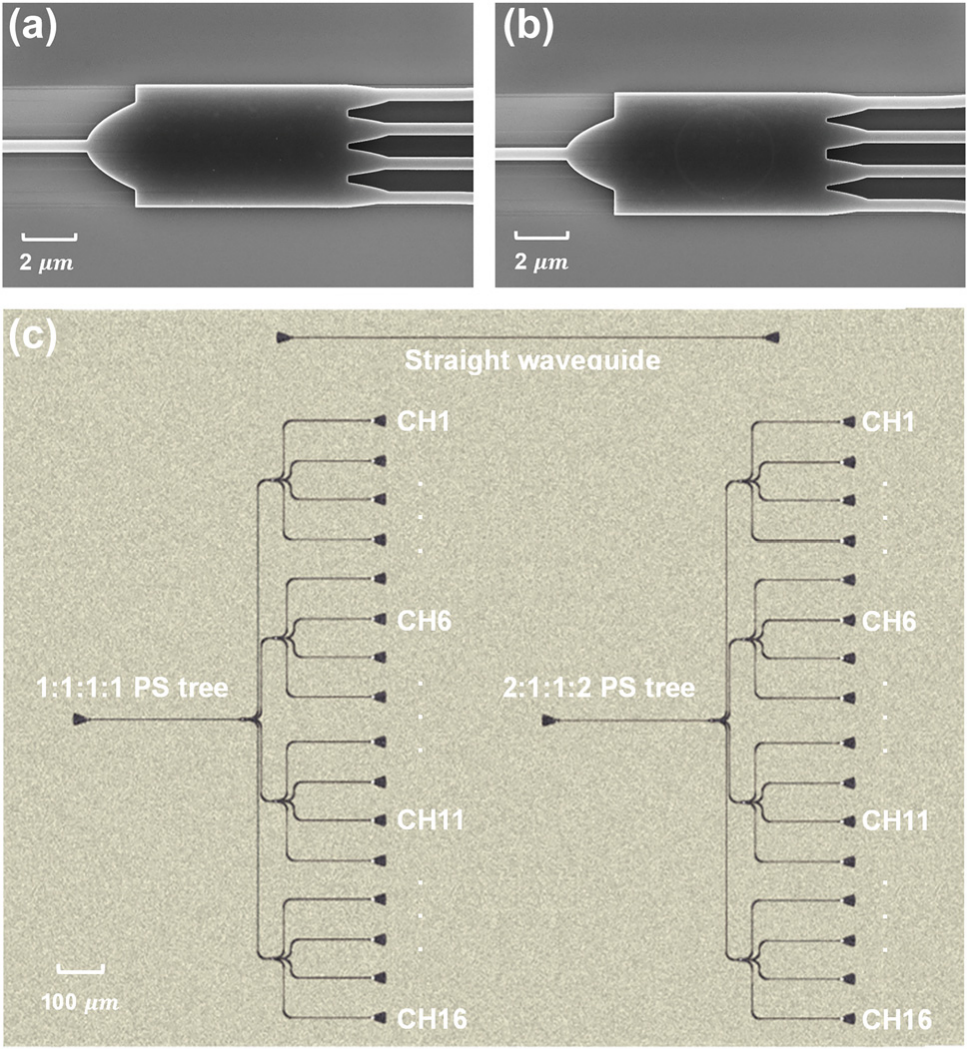
SEM images of (a) PS-1 and (b) PS-2. (c) The test pattern of PS trees and a straight waveguide.

The optimized PSs were fabricated on the SOI platform provided by Applied Nanotools Inc. The silicon substrate has a thickness of 725 nm, and a 220-nm silicon device layer is patterned on top of a 2-μm buried oxide layer, using 100 keV electron beam lithography technology. The patterned devices were fully etched using an e-beam mask material and an anisotropic ICP-RIE etching process. A 2.2-μm cladding oxide layer was deposited onto the silicon device layer using a plasma-enhanced chemical vapor deposition (PECVD) process. The fabricated PSs can be seen in Figure 4(a) and (b). The minimum feature size of PSs is larger than 150 nm, and both have a compact footprint of 12.32 × 5 μm^2^.

### 3.2 Defining performance metrics

To characterize the fabricated power splitter devices, performance metrics are defined and calculated in this section. First, the insertion loss (IL) of a single device is defined as follows:

(6) $$IL=-10\log\left[\frac{\overset{16}{\underset{k=1}{\sum }}P_{\text{out,k}}}{P_{in}}\right]/2,$$

where *P*_out,*k*_ represents the normalized power measured at the *k*-th output channel of the two-stage PS tree. As mentioned earlier, two-stage PS trees were used in the measurement procedure. Therefore, the total loss of the PS tree was divided to calculate the IL of a single device by the number of stages, which is 2 in this study. Next, to express how evenly the PS-1 distributes light to its 4 output ports, the imbalance (IM) is defined as follows:

(7) $$IM=-10\log\left[\frac{\min\left(P_{\text{out,}1},P_{\text{out,}6},P_{\text{out,}11},P_{\text{out,}16}\right)}{\max\left(P_{\text{out,}1},P_{\text{out,}6},P_{\text{out,}11},P_{\text{out,}16}\right)}\right]/2.$$

Here, the 1st output channel is connected exclusively through the 1st ports of two PSs. Similarly, the 6th, 11th, and 16th channels involve only 2nd, 3rd, and 4th ports of two PSs, respectively. In other words, the 1st, 6th, 11th, and 16th output channels represent each identical output port of a single device.

For the PS-2, the objective is not to evenly distribute light to the four output ports, so evaluating the performance of the device using IM is not appropriate. Therefore, a performance metric called the targeted imbalance (TIM) has been defined. First, let’s define the TIM between an arbitrary *i*-th output port and *j*-th output port as follows:

(8) $$\begin{array}{ll} TIM_{i,j} & =\left|\log\left[\frac{P_{\text{out,i}}/P_{\text{out,j}}}{R_{i,j}}\right]/2\right|\\ & \;\;\;\;\text{for}\;i,j=1,6,11,16\;\text{and}\;i<j, \end{array}$$

where *R*_*ij*_ represents the ideal output ratio between the *i*-th and *j*-th output channels. The ideal output ratio between the 1st and 6th output ports is 4; thus, for the case of *i* = 1 and *j* = 6, *R*_1,6_ becomes 4. TIM allows us to numerically express how close the measured power splitting ratio is to the desired power splitting ratio. With TIM defined for specific pairs of output ports, the overall TIM for the device can be defined as follows:

(9) $$TIM=\max\left(TIM_{1,6},TIM_{1,11},\ldots ,TIM_{11,16}\right).$$

When the defined TIM is applied to the PS-1, *R*_*i*,*j*_ becomes 1 for all *i* and *j*, resulting in TIM being equal to IM. Therefore, TIM can be considered a generalized expression of IM. Like the IL case, IM and TIM were divided by the number of stages.

### 3.3 Experimental results

The transmission of the PSs was measured using the PS trees shown in Figure 4(c). To enhance the reliability of the measurements, a two-stage PS tree test pattern was utilized [30]. Initially, light generated from a tunable laser passed through a polarization controller to ensure the generation of the fundamental TE mode. Subsequently, it was coupled into the waveguide via TE-type grating couplers and split into four output ports by the first-stage PS. Each output port from the first-stage PS was then connected to the second-stage PSs, where it was split into four branches again. Finally, the light is divided into 16 output channels and emitted to free space through TE-type grating couplers. Then the output power was measured using external photodetectors. The losses of the waveguide, input and output grating couplers are included in the measured power. Therefore, the measured transmission was normalized by subtracting the loss from the reference waveguide including input and output grating couplers.

**Figure 5:**
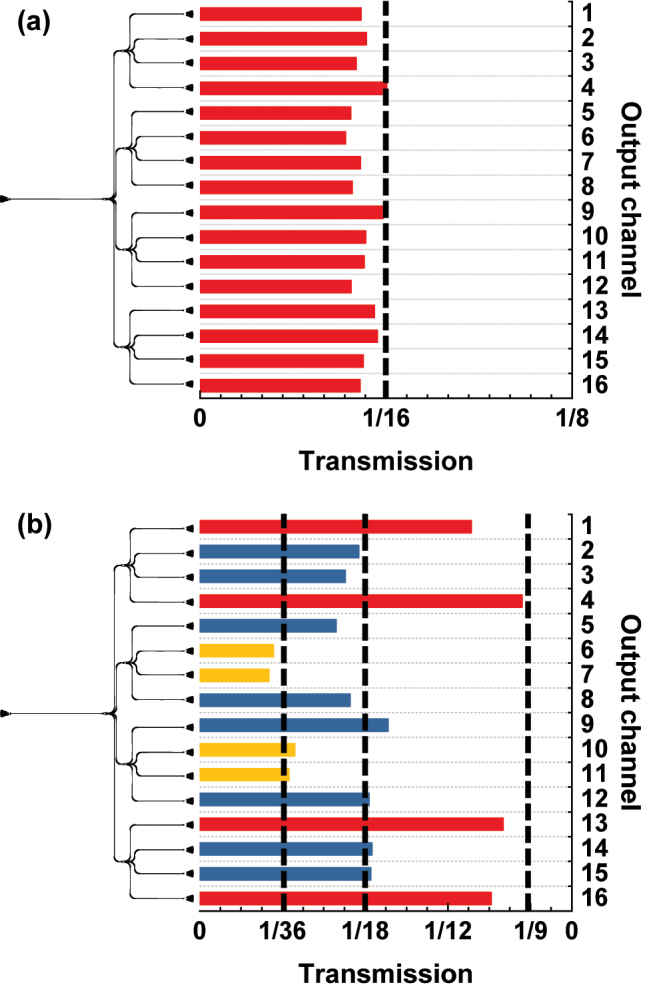
Transmission of 16 output channels in the (a) PS-1 tree, and (b) PS-2 tree at the wavelength of 1,550 nm. Transmission is represented as a ratio of output power over input power, not in dB scale. The desired output transmissions are marked with black dashed lines. The output channels targeting 1/9 of the input power are colored in red, 1/18 in blue, and 1/36 in yellow.

**Figure 6:**
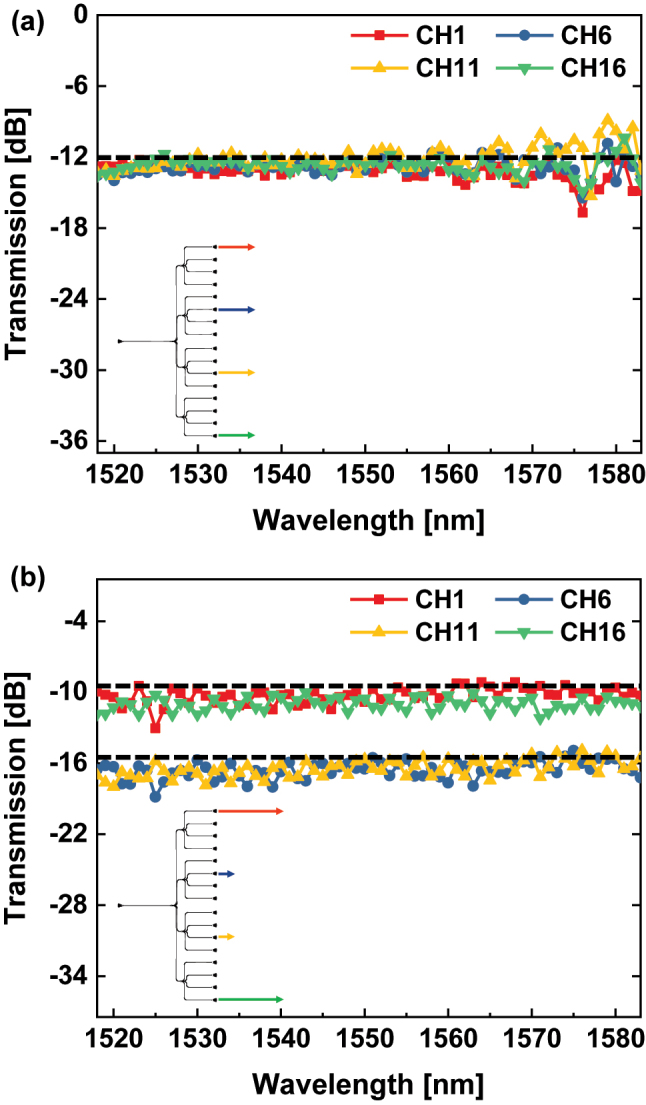
Transmission of the 1st, 6th, 11th, and 16th output channels in the (a) PS-1 tree, and (b) PS-2 tree as a function of wavelength. The desired output transmissions are marked with black dashed lines. In the PS-1 tree, the ideal transmission of all output channels is −12.04 dB. In the PS-2 tree, the ideal transmission of the 1st, and 16th output channels is −9.54 dB, while the ideal transmission of the 6th, and 11th output channels is −15.56 dB.

**Figure 7:**
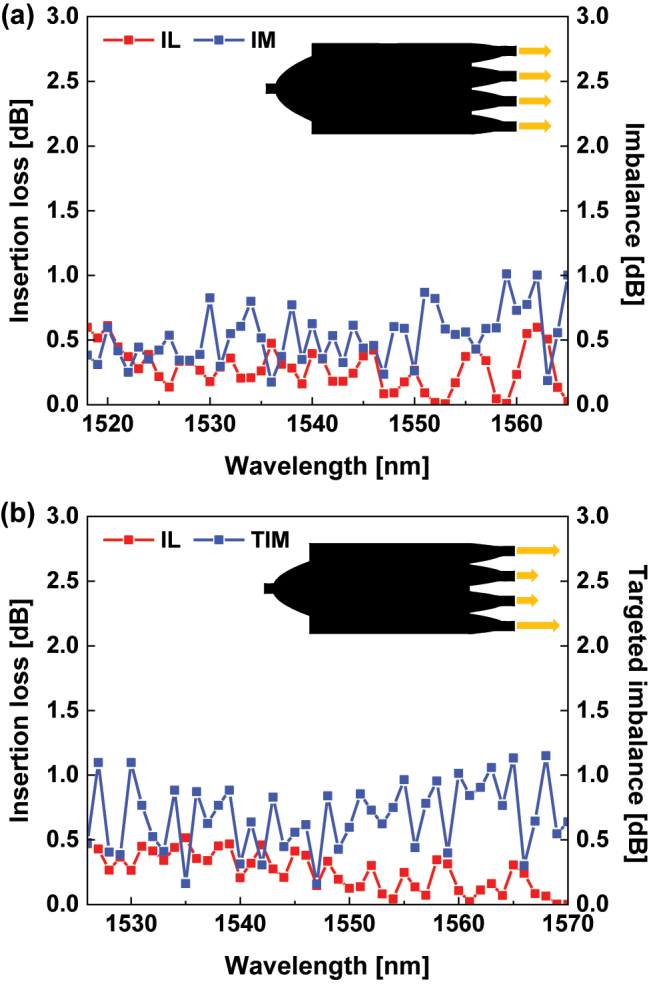
Performance metrics of proposed PSs. (a) Average insertion loss and imbalance of the single PS-1. (b) Average insertion loss and targeted imbalance of the single PS-2.

The transmissions of output channels in the PS trees are illustrated in Figures 5 and 6. Figure 5 shows that the 16 output channels of both PS trees achieved the desired splitting ratios at the wavelength of 1,550 nm. The input power was evenly distributed among the 16 output channels in the PS-1 tree. On the other hand, the PS-2 is targeted to an unequal splitting ratio, so the input power was split to each output channel with different proportions. In Figure 5(b), output channels aiming for 1/9 of the input light are colored in red, 1/18 in blue, and 1/36 in yellow. Transmissions of the four output channels consisting only of identical ports in the PS trees are illustrated in Figure 6. Due to the limitations of the experimental equipment, only the wavelength range from 1,518 nm to 1,583 nm was measured. It can be observed that achieving efficient power splitting at predetermined ratios across a wide range of wavelengths. Bandwidths of PSs were determined based on these four output transmissions in Figure 6. The IL, IM, and TIM were calculated with the definition in Section 3.2. The performance metrics of the single PS are shown in Figure 7. The PS-1 shows a low IL of less than 0.61 dB and a low IM of less than 1.01 dB in the bandwidth of 1,518–1,565 nm. In the case of PS-2, a low IL of less than 0.52 dB and a low TIM of less than 1.15 dB were obtained within the bandwidth of 1,526–1,570 nm.

Although measurement error is reduced by two-stage PS trees, the performance of PSs deviated slightly from the intended design due to fabrication error. The comparison of our 1 × 4 PS and previous literature results is displayed in Table 1. The PSs proposed in this paper have relatively low insertion losses and broad bandwidths with compact footprints. Also, the PS-1 and PS-2 show competitive imbalance and targeted imbalance, respectively.

**Table 1:** Comparison of the 1 × 4 PS proposed in this paper with other literature results.

Structure	Splitting ratio	IL [dB]	IM [dB]	Bandwidth [nm]	Footprint [μm^2^]
MMI [17]	1:1:1:1	<0.62	<0.89	1,520–1,624	36 × 6
MMI [18]	1:1:1:1	<0.76	<0.84	1,555–1,570	8.14 × 12
MMI [18]	1:1:1:1	<1.08	<0.81	1,545–1,560	6 × 7.2
MMI [29]	1:1:1:1	<1.0	–	1,530–1,565	8.2 × 3.2
Inverse taper [30]	1:1:1:1	<0.4	<0.68	1,510–1,550	12.5 × 6
MMI (this work)	1:1:1:1	<0.61	<1.01	1,518–1,565	12.32 × 5
MMI (this work)	2:1:1:2	<0.52	<1.15	1,526–1,570	12.32 × 5

## 4 Discussion and conclusion

In this work, we present two types of 1 × 4 PSs that distribute light at different power ratios. The experimental results show that both PSs perform power splitting with accurate ratios, low losses, broad bandwidths, and compact footprints. Numerical analysis identified suitable taper configurations for MMI-based splitters, leading to the selection of parabolic tapers. The PSs were inverse-designed using PSO. The dimensions of the front and rear parts were optimized sequentially to reduce optimization complexity and prevent local minima problems. The designed PSs can be fabricated using a CMOS-compatible fabrication process and implemented on a 220-nm SOI platform. The fabricated PSs have a compact footprint of 12.32 × 5 μm^2^, and the minimum feature size of the devices is larger than 150 nm. Even though these PSs were fabricated with e-beam lithography, both devices can be fabricated with deep ultra-violet (DUV) lithography which can support mass production. In the experiment, two-stage PS trees were measured to enhance reliability. The PS-1 exhibited an IL of less than 0.61 dB and an IM of less than 1.01 dB within the bandwidth of 1,518–1,565 nm. Similarly, the PS-2 showed an IL of less than 0.52 dB and a TIM of less than 1.15 dB within the bandwidth of 1,526–1,570 nm. Given the advantages of high performance and compactness in our proposed 1 × 4 PSs, we expect them to be widely used in high-performance and highly integrated PICs. Additionally, leveraging the excellent performance of parabolic input tapers in MMI-based PSs, we anticipate designing multi-output PSs with more than four output ports. However, an increase in the number of output ports leads to an increase in footprint as well as in design parameters, so it is necessary to conduct inverse design with consideration of computational cost. Furthermore, we suggest the possibility of designing PSs with arbitrary splitting ratios by varying the width of the parabolic input taper.
